# A logistic analysis prediction model of TMJ condylar erosion in patients with TMJ arthralgia

**DOI:** 10.1186/s12903-021-01687-w

**Published:** 2021-07-24

**Authors:** Rüdiger Emshoff, Annika Bertram, Linus Hupp, Ansgar Rudisch

**Affiliations:** 1 Orofacial Pain and TMD Unit, University Clinic of Oral and Maxillofacial Surgery, Medical University of Innsbruck, Anichstraße 35, 6020 Innsbruck, Austria; 2 Otto Von Guericke University of Magdeburg, Magdeburg, Germany; 3 University Clinic of Oral and Maxillofacial Surgery, Medical University of Innsbruck, Innsbruck, Austria; 4 University Clinic of Radiology, Medical University of Innsbruck, Innsbruck, Austria

**Keywords:** Temporomandibular joint, Arthralgia, Magnetic resonance imaging, Disk displacement, Condylar erosion, Bone marrow edema, Predictive modeling

## Abstract

**Background:**

In terms of diagnostic and therapeutic management, clinicians should adequately address the frequent aspects of temporomandibular joint (TMJ) osteoarthritis (OA) associated with disk displacement. Condylar erosion (CE) is considered an inflammatory subset of OA and is regarded as a sign of progressive OA changes potentially contributing to changes in dentofacial morphology or limited mandibular growth. The purpose of this study was to establish a risk prediction model of CE by a multivariate logistic regression analysis to predict the individual risk of CE in TMJ arthralgia. It was hypothesized that there was a closer association between CE and magnetic resonance imaging (MRI) indicators.

**Methods:**

This retrospective paired-design study enrolled 124 consecutive TMJ pain patients and analyzed the clinical and TMJ-related MRI data in predicting CE. TMJ pain patients were categorized according to the research diagnostic criteria for temporomandibular disorders (RDC/TMD) Axis I protocol. Each patient underwent MRI examination of both TMJs, 1–7 days following clinical examination.

**Results:**

In the univariate analysis analyses, 9 influencing factors were related to CE, of which the following 4 as predictors determined the binary multivariate logistic regression model: missing posterior teeth (odds ratio [OR] = 1.42; *P* = 0.018), RDC/TMD of arthralgia coexistant with disk displacement without reduction with limited opening (DDwoR/wLO) (OR = 3.30, *P* = 0.007), MRI finding of disk displacement without reduction (OR = 10.96, *P* < 0.001), and MRI finding of bone marrow edema (OR = 11.97, *P* < 0.001). The model had statistical significance (chi-square = 148.239, Nagelkerke R square = 0.612, *P* < 0.001). Out of the TMJs, 83.9% were correctly predicted to be CE cases or Non-CE cases with a sensitivity of 81.4% and a specificity of 85.2%. The area under the receiver operating characteristic curve was 0.916.

**Conclusion:**

The established prediction model using the risk factors of TMJ arthralgia may be useful for predicting the risk of CE. The data suggest MRI indicators as dominant factors in the definition of CE. Further research is needed to improve the model, and confirm the validity and reliability of the model.

## Background

Osteoarthritis (OA) of the temporomandibular joint (TMJ) is a degenerative process characterized by deterioration of articular tissue with concomitant radiographically detectable osseous changes including flattening, sclerosis, osteophytes, and erosion involving the condyle and/or articular eminence [1–3]. The prevalence of TMJ OA is estimated to range from 8 to 35% in the general population based on radiographic assessment [4, 5]. The clinical diagnosis of TMJ OA depends on clinical features such as joint pain and crepitus noises [6, 7]. Patients may experience prolonged pain and disability which causes chronic symptoms becoming more refractory to traditional medical treatment approaches [8, 9].

It is now thought that the underlying mechanisms contributing significantly to the initiation and development of OA are synovial inflammation and increased subchondral bone turnover [10, 11]. Increased bone remodeling is thought to characterize OA progression, with early bone loss accompanied by remodeling, subchondral bone sclerosis and full cartilage loss [12].

More recently, erosive alterations in TMJ OA has become a focus of interest. The pathophysiology of erosive alterations is unclear and the question whether erosive OA is as a separate entity or a more severe form of TMJ OA remains unresolved. Condylar erosion (CE) of the TMJ involves articular cartilage and adjacent cortical and subcortical bone structures [13, 14]. CE is understood as an inflammatory subset of OA, considered as a sign of progressive OA [14, 15], and has been linked to characteristic clinical findings, including irregular or deviating jaw function, joint sounds, and pain [15, 16]. Further, CE is associated with magnetic resonance imaging (MRI) findings of disk displacement [17, 18] and should be adequately addressed in terms of diagnostic and therapeutic management to prevent changes in dentofacial morphology or limited mandibular growth [19].

For the evaluation of structural TMJ OA characteristics, computed tomography [20, 21] and cone beam computed tomography (CBCT) [22, 23] are currently considered as the gold standard. OA, however, is currently recognized as an entire joint failure [24], i.e., MRI allowing multiplanar visualization of all the joint components has been increasingly used in TMJ OA and inflammatory joint diseases as an outcome measure [25, 26]. Effusion and bone marrow lesions are acknowledged as important markers for symptomatology and prognosis in both TMJ rheumatoid arthritis and OA.

To the best of the authors’ knowledge, there are no studies available addressing the role of clinical and imaging parameters in the definition of TMJ CE in a multivariate design. Thus, the aim of the present study was to identify the most associated variables for prediction of TMJ CE in patients with TMD pain according to clinical parameters and magnetic MRI findings.

## Methods

### Study design, population, inclusion and exclusion criteria

The study group, selected over a period of approximately 20 years, consisted of 124 consecutive patients with a unilateral TMJ pain condition of arthralgia. There were 222 females and 26 males, aged between 18 and 67 years with a mean age of 37.5 years. All participants had initially been referred to, and were examined at the Orofacial Pain Unit, Department of Oral and Maxillofacial Surgery, Medical University of Innsbruck.

The subjects were informed about the study procedure, and informed consent was received from each participant. This retrospective study followed the medical protocols and ethics outlined in the Declaration of Helsinki. Given the retrospective nature of this study, ethical approval of the study was waived by the Ethics Committee of the Medical University of Innsbruck.

Clinical data, consisting of demographic, clinical and treatment records were collected from chart records. The clinical assessment was performed by one clinician (RE) specialized in temporomandibular disorders (TMD) and orofacial pain. Each subject completed a visual pain rating to assess severity of pain by using a 100-mm visual analogue scale, ranging from 0 (no pain) to 100 (very severe pain), i.e. patients registered the mean pain perceived in the last seven days. This scale has been used extensively in randomized trials and has shown good construct validity in comparison with other pain measures [27, 28]. Indications for the MRI of each subject at baseline were retrieved from clinical records.

The inclusion consisted of (i) a unilateral Research Diagnostic Critera (RDC) for TMD diagnosis of unilateral arthralgia, (ii) a pain duration of at least 1 month, (iii) a pain intensity of at least 10 mm as assessed by a VAS, (iv) an age between 18–70 years, (v) in cases of tooth loss, a posterior tooth missing for more than 6 months, and (vi) MRI performed within 2 weeks after the clinical examination. The exclusion criteria included (i) a RDC/TMD diagnois of myofacial pain with limited mouth opening, (ii) a RDC/TMD diagnosis of disk displacement with reduction, (iii) a cervical or head pain, (iv) an acute head or neck infection, (v) a diagnosis of a collagen vascular disease, (vi) a previous head or neck trauma in history, (vii) a history of previous TMD treatment, and (viii) a diagnosis of a debilitating mental or physical illness.

### MRI data acquisition

MRI was carried out with a 1.5 T MR scanner (Vision, Siemens AG, Erlangen, Germany) and a dedicated circular-polarized transmit-and-receive TMJ coil. The data were collected on a 252 × 256 matrix with a field of view of 145 mm giving a pixel size of 0.60 × 0.57 mm. With the patient in a supine position, 15 paracoronal and 8 para-sagittal slices were obtained of each TMJ using a TSE (turbo spin echo)-PD (proton density) sequence (repitition time of 2800 ms, echo time of 15 ms) and a TIRM (turbo inversion recovery magnitude) sequence (repitition time of 4000 ms, echo time of 30 ms, inversion time of 150 ms) with thin slices of 3 mm. MR images were corrected to the horizontal angulation of the long axis of the condyle [29].

**Fig. 1:**
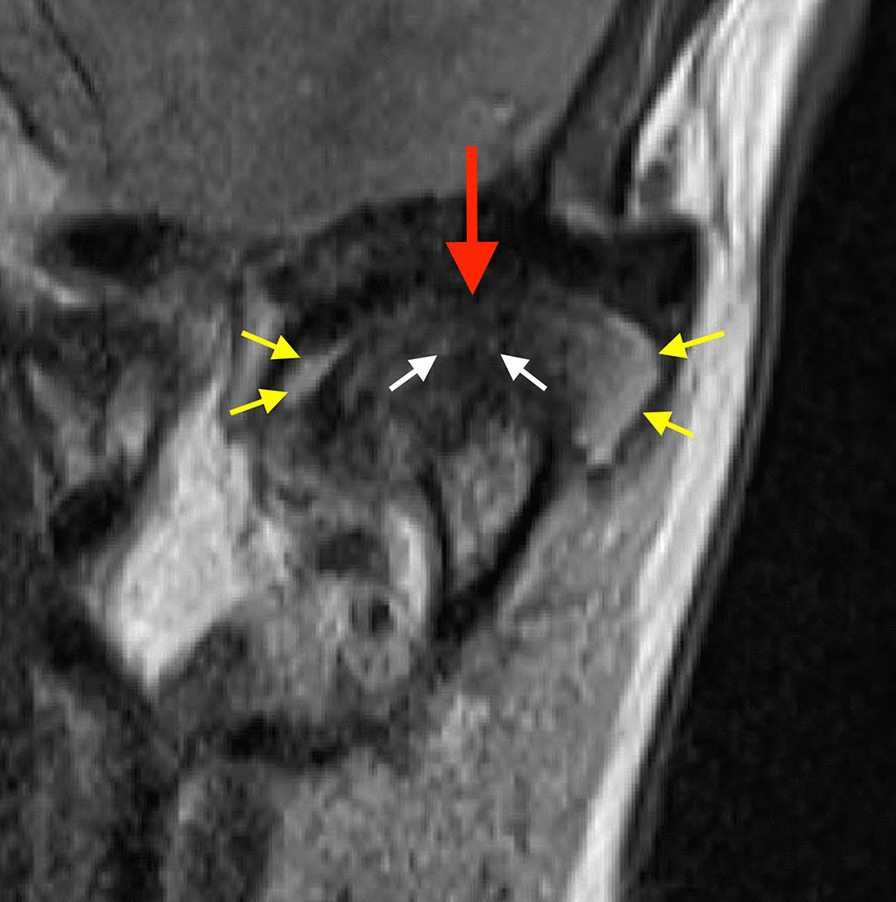
Closed–mouth-related MR images in a 61-year-old female with a 18-month history of left TMJ pain, a TMJ pain-side-related clinical diagnosis of TMJ AR, and the MRI finding of disk displacement without reduction, CE, bone marrow edema, and effusion. Coronal MR image shows condyle with CE (red arrow), bone marrow edema (white arrows), and effusion in the superior joint compartment (yellow errors)

**Fig. 2:**
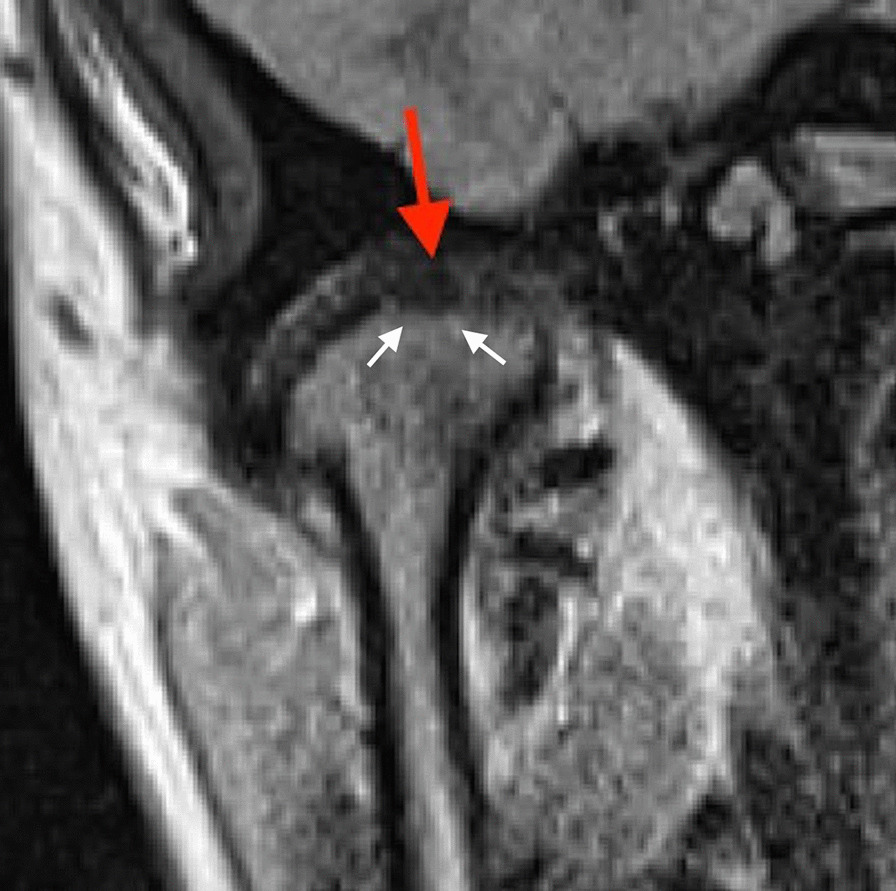
Closed–mouth-related MR images in a 18-year-old female with a 12-month- history of right TMJ pain, a TMJ pain-side-related clinical diagnosis of TMJ ‘AR and  disk displacement without reduction with limited opening (DDwoR/wLO)’, and the MRI finding of disk displacement without reduction and CE. Coronal MR image shows condyle with CE (red arrow) and bone marrow edema (white error)

Each subject received an individual nonferromagnetic intermaxillary device to obtain the different mouth opening positions. Sequential bilateral T1- and T2- weighted images were made at the closed mouth and the respective maximum mouth opening positions. Those T1-weighted images were selected for analysis of the disk-condyle relationship that depicted the disk, condyle, articular eminence, and glenoid fossa. Normal disk position was defined by location of the posterior band of the disk at the superior or 12 o’clock position relative to the condyle, whereas disk displacement was defined as the posterior band of the disk being in an anterior, anteromedial, anterolateral, medial, or lateral position relative to the superior part of the condyle. Diagnosis of TMJ disk-condyle relationship was categorized as normal and disk displacement with and without reduction, and defined according to the finding of a closed mouth-related diagnosis of absence or presence of disk displacement associated with or without an open mouth-related interposition of the disk between the condyle and the articular eminence [30]. MRI diagnosis of condylar osteoarthrosis was defined by the presence of subchondral sclerosis, erosion and osteophyte [29, 31]. MRI diagnosis of CE was defined as an interruption or absence of the cortical lining of the condyle [32, 33]. On the T2-weighted images, joint effusion was identified as an area of high signal intensity in the region of the joint space. When more than a line of high signal was evident in at least 2 consecutive sections, it was considered positive for TMJ effusion [34]. Bone marrow edema was defined by the presence of a hypointense signal on T1-weighted images and a hyperintense signal on T2-weighted images (Figs. 1 and 2) [35].

Reliability scores were determined by administering the imaging criteria on a set group of images, thereby allowing for intrarater comparison. The intraobserver reliability was strong (Κappa > 0.85) to exellent (Κappa = 1.00) for all of the MRI diagnoses.

### Data analysis

Sample size was established at 239 TMJs based on the following assumptions: the prevalence of CE among TMJ arthralgia patients would be 40%, and 15% among asymptomatic controls [36, 37], with an alpha error of 0.05, and a statistical power of 95%. For sample size estimation, the G*Power software (version 3.1) was applied.

Categorical variables were analysed by the chi-square test, and continuous variables were expressed as mean ± SD with an independent-samples *t* test. Stepwise logistic regression analyses were performed to identify multivariate predictors of CE. Based on previous studies, the variables, which were included in the model, were age (years), gender, time since pain onset, pain intensity, the number of missing posterior teeth, bone marrow edema, and effusion [14, 38]. Significance was set at *P* < 0.05. For the statistical analysis, the PASW 27.0 (SPSS Statistics, IBM, Chicago) package was used.

A receiver operating characteristic (ROC) curve was used to describe the discrimination abilities of the predictive indicators. The area under the curve (AUC) provides a global summary statistic of test accuracy, and guidelines suggest that 0.5 < AUC ≤ 0.7 represent low accuracy, 0.7 < AUC ≤ 0.9 moderate accuracy, and 0.9 < AUC ≤ 1.0 represents high accuracy. An AUC above 0.75 is considered as good. The 95% confidence interval (CI) was calculated, and *P* < 0.05 was considered to indicate statistical significance. For the statistical analysis, the PASW 27.0 (SPSS Statistics, IBM, Chicago) package was used.

## Results

At least 1 TMJ with CE was present in 61.3% of the 124 patients with TMJ arthralgia. CE was found in 50.0% of the TMJs with arthralgia, and in 25.8% of TMJs without pain. Analysis of side-related data showed CE (50.0% vs 25.8%) (*P* < 0.001) to be more prevalent in TMJs with arthralgia than in those without pain (Table 1).

**Table 1:** Condylar erosion by subject and TMJ side demonstrated by MRI

Distribution of CE	Subjects (n = 124)	TMJs		
		TMJs with arthralgia (n = 124)	TMJs without pain (n = 124)	Total (n = 248)
Presence of CE				
TMJ arthralgia side only (n) (%)	44 (35.5)	44 (71.0)	–	44 (17.7)
TMJ non-pain side only (n) (%)	14 (11.3)	–	14 (43.8)	14 (5.7)
TMJ arthralgia and non-pain side (n) (%)	18 (14.5)	18 (29.0)	18 (56.3)	36 (14.5)
Total (n) (%)	76 (61.3)	62 (50.0)	32 (25.8)	94 (37.9)^a^

*CE* condylar erosion, *TMJ* temporomandibular joint, *MRI* magnetic resonance imaging, *n* number of TMJs, *(%)* percent

^a^*P* < 0.0001, based on chi-squared test

The clinical and MRI indicators assessed in this study are listed in Table 2. Nine indicators were significantly different between the CE and non-CE groups: missing posterior teeth (*P* < 0.001), pain intensity (*P* = 0.002), RDC/TMD of arthralgia (*P*  = 0.004), RDC/TMD of arthralgia coexistant with DDwoR/wLO (*P* < 0.001), and MRI findings of disk displacement with reduction (*P*  = 0.001), disk displacement without reduction (*P*  = 0.001), subchondral sclerosis (*P*  = 0.001), bone marrow edema (*P* < 0.001), and effusion (*P* = 0.001).

**Table 2:** Clinical and MRI indicators to predict condylar erosion of the TMJ

Predictors	TMJs with condylar erosion (n = 94)	TMJs without condylar erosion (n = 154)	Total (n = 248)	*P* Value
Clinical items				
Age (years) (mean ± SD)	39.0 (14.4)	36.6 (12.9)	37.5 (13.5)^a^	0.174
Gender (n) (% female)	88 (93.6)	134 (87.0)	222 (89.5)^b^	0.073
Missing posterior teeth (mean)	1.56	0.78	0.124^a^	< 0.001
Time since pain onset (weeks) (mean ± SD)	13.9 (26.0)	10.8 (27.1)	12.0 (26.7)^a^	0.377
Pain intensity (mm) (mean ± SD)	32.3 (32.3)	20.2 (28.7)	24.8 (30.6)^a^	0.002
TMD/RDC diagnosis				
Arthralgia (n) (%)	24 (25.5)	44 (28.6)	66 (26.6)^b^	0.004
Arthralgia with DDwoR/wLO (n) (%)	38 (40.4)	18 (11.7)	56 (22.6)^b^	< 0.001
MRI items				
Disk displacement with reduction (n) (%)	20 (21.3)	64 (41.6)	48 (33.9)^b^	0.001
Disk displacement without reduction (n) (%)	70 (74.5)	25 (16.2)	95 (38.3)^b^	0.001
Subchondral sclerosis (n) (%)	38 (40.4)	96 (62.3)	134 (54.0)^b^	0.001
Osteophyte (n) (%)	30 (31.9)	33 (21.4)	63 (25.4)^b^	0.066
Bone marrow edema (n) (%)	61 (69.4)	20 (13.0)	48 (32.7)^b^	< 0.001
Effusion (n) (%)	28 (29.8)	19 (12.3)	47 (19.0)^b^	0.001

*TMJ* temporomandibular joint, *MRI* magnetic resonance imaging, *SD* standard deviation, *(%)* percent, *n* number of TMJs

^a^Based on independent samples *t* test

^b^Based on chi-squared test

Using binary multivariate logistic analysis, 4 risk factors were identified that correlated best as predictors of CE: missing posterior teeth (odds ratio [OR] = 1.42; *P* = 0.018), RDC/TMD of arthralgia coexistant with DDwoR/wLO  (OR = 3.30, *P* = 0.007), MRI finding of disk displacement without reduction (OR = 10.96, *P* < 0.001), and MRI finding of bone marrow edema (OR 11.97, *P* < 0.001). Based on the modeling study, the probability of CE was calculated according to the following equation: *P* (CE) = 1 / (1 + exp (ODDS)), where ODDS =  − 3.129 + 0.352 missing posterior teeth + 0.686 DDwoR/wLO + 2.234 disk displacement without reduction + 2.482 bone marrow edema (Table 3).

**Table 3:** Results of binary multivariate logistic regression analysis of influencing factors of condylar erosion of the TMJ

Predictors	B	S.E	Wald statistic	*P*	Odds ratio	95% CI
Clinical items						
Missing posterior teeth (mean ± SD)	0.352	0.149	5.574	0.018	1.422	1.062–1.906
TMD/RDC diagnosis						
Arthralgia with DDwoR/wLO (n) (%)	0.686	0.445	7.198	0.007	3.300	1.379–7.894
MRI items						
Disk displacement without reduction (n) (%)	2.234	0.392	37.414	< 0.001	10.959	5.087–23.607
Bone marrow edema (n) (%)	2.482	0.406	33.876	< 0.001	11.968	5.400–26.488

*TMJ* temporomandibular joint, *MRI* magnetic resonance imaging, *SD* standard deviation, *(%)* percent, *n* number of TMJs

**Fig. 3:**
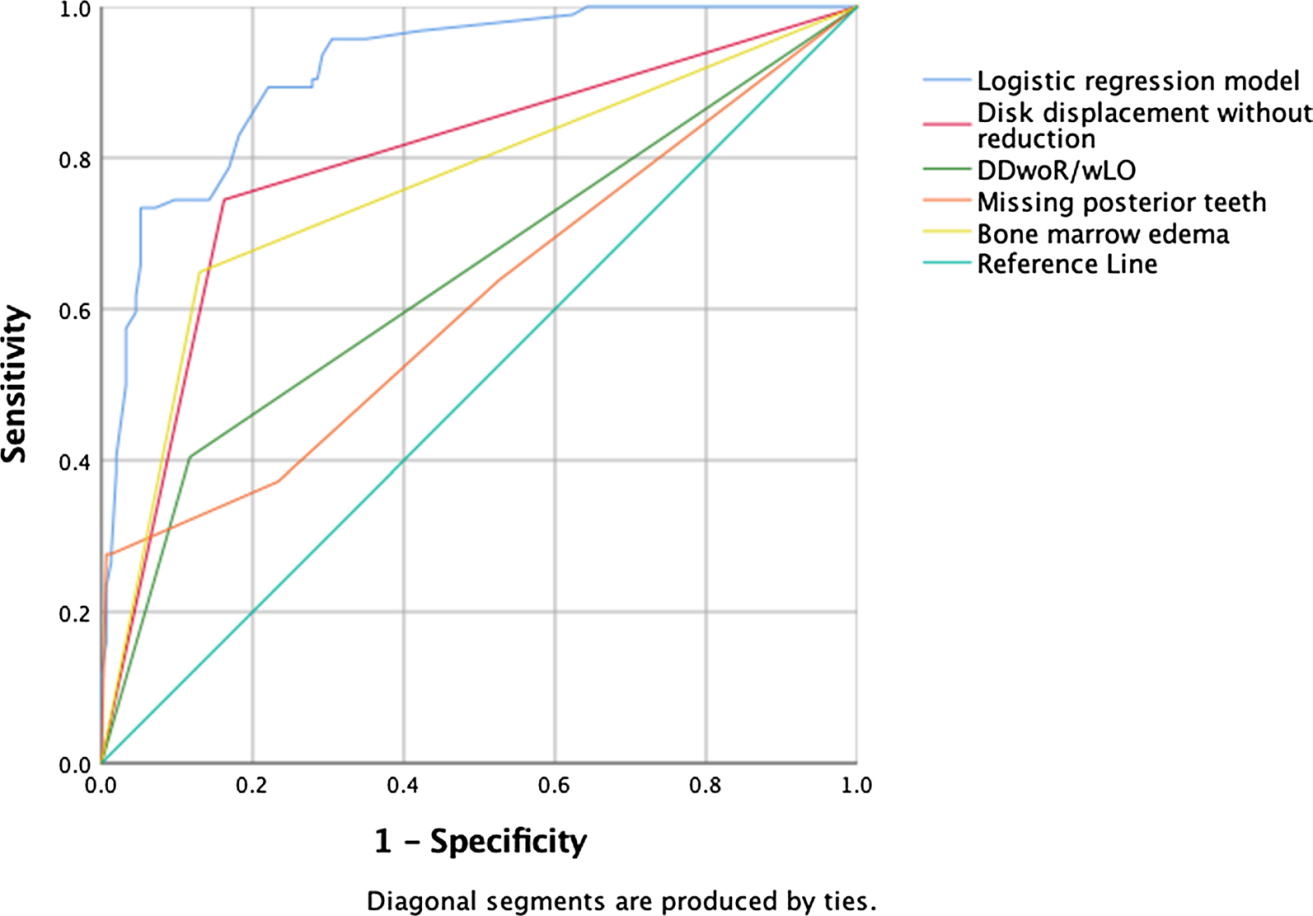
Receiver operating characteristic (ROC) curves of multivariate logistic regression model illustrating the predictive capacity of the model. The area under the curve of ROC curves of individual variables, namely MRI finding of disk displacement without reduction, RDC/TMD of arthralgia coexistant with disk displacement without reduction with limited mouth opening (DDwoR/wOL), missing posterior teeth, and bone marrow edema

ROC curves of each independent variable, and the multivariate logistic model are plotted in Fig. 3. The AUC demonstrated statistical significance and a high diagnostic value for the logistic model (AUC = 0.916, SE = 0.018, *P* < 0.001, 95% CI: 0.885 to 0.954). There was an overall prediction accuracy of 83.9% using multivariate logistic function when classification was determined using all data points (Table 4). The rates of correct prediction were 81.4% for the CE cases (sensitivity) and 85.2% for the controls (specificity), and the positive and negative predictive values were 74.5 and 89.6%, respectively.

**Table 4:** Percentages of correct classifications of TMJs conducted by binary multivariate logistic regression analysis

	Predicted group membership		
Actual classification	Non-condylar erosion (%)	Condylar erosion (%)	Total
Non-condylar erosion	138 (87.0)	16 (13.0)	154
Condylar erosion	24 (23.9)	70 (76.6)	94
Total	156	92	248

*TMJ* temporomandibular joint, *(%) percent,* 83.9% of original grouped cases correctly classified

## Discussion

The results of MRI in the present study showed TMJ arthralgia patients to be associated with a high rate of CE (61.3%). These findings compare favourably with the observations of other studies reporting TMJ CE to occur in TMJ pain patients with frequencies ranging from 34 to 85% [15, 39, 40], whereas in asymptomatic subjects rates are described to range from 0% to 7.4% [15, 37, 39]. Concerning the observed prevalence rates of CE (50%) in TMJs with arthralgia, the findings may correspond to those of previous research reports describing prevalences CBCT findings of CE in TMJ arthralgia with 60% [16], and in TMJ arthritis with 94% [2]; the frequencies in asymptomatic TMJs being described with ranges from 6% [30] to 21% [16]. However, the results may be not directly comparable as in these studies confounding variables were not considered in a multivariate design, i.e. studies failed to take into account relevant clinical and MRI parameters simultaneously.

To the best of our knowledge, this is the first study to provide relative odds for the estimation of CE of the TMJ in a multivariate design using logistic regression techniques for analysis. It provides a perspective to the contribution of clinical and MRI parameters to the occurrence of a CE. While the clinical parameters of missing posterior teeth (1: 1.42), and RDC/TMD of arthralgia coexistant with DDwoR/wOL (1: 3.30) contributed a minor to moderate amount to the change in risk, a clear definition of the CE group was evident for the MRI variables of disk displacement without reduction (1: 10.96), and bone marrow edema (1: 11.97). Therefore, based on this study, the presence of MRI findings of disk displacement without reduction and bone marrow edema may be considered a dominant factor in the definition of CE of the TMJ. Considering the aspect of arthritic TMJ conditions as an underlying mechanism in the development of changes in dentofacial morphology or limited mandibular growth [41, 42], further investigations are indicated to clarify which additional clinical and/or MRI variables may be associated with an elevated risk for arthritic CE signs of TMJ OA, while only a prospective cohort study rather than a case–control study will estimate the etiologic contribution of defined variables to CE.

Most studies have focused on the identification of risk factors of clinical TMD diagnoses [34, 38], while an effective risk prediction tool for CE is lacking. In the current research, CE was linked to 9 factors screened by multivariate logistic regression analysis, but only 4 factors used as predictors were included in the regression analysis. Selected predictors, i.e., missing posterior teeth, RDC/TMD of arthralgia coexisting with DDwoR/wLO, and MRI findings of disk displacement without reduction and bone marrow edema were significantly correlated with CE, a finding which is consistent with previous research [43–46].

In classifying 83.9% of participants, with an AUC of 0.916, the prediction model developed by multivariate regression analysis was efficient. In order to classify the high-risk population with active condylar alterations in the first clinical environment, the prediction model can be used as a risk prediction method for CE, and thereby provide valuable information for further clinical follow-up and treatment approaches.

If patients are identified that they are at a high risk by this prediction, clinicians may be able to control some important risk factors to reduce the risk of active progressive condylar alterations. In practice, considering a hypothetical case, involving a patient with TMJ arthralgia, where the number of missing posterior teeth = 4, DDwoR/wLO = 0, disk displacement without reduction = 1, and bone marrow edema each = 1, the odds prediction equation is ODDS = exp(− 3.129 + 0.352 × 4 + 0.686 × 0 + 2.234 × 1 + 2.482 × 1), and the *P* (CE) = 0.952. That is, the model predicts that 95% of the TMJs may be at risk for the occurrence of CE.

Several factors are acknowledged as risk items for the development of CE [14, 47, 48]. They comprise gender, age systemic, hormonal factors, and arthritis [49, 50]. Mechanical factors include disk displacement, occlusion, trauma, and increased friction at the joint [51, 52]. Further, overloading is the main unterlying disorder in any synovial joint, including the TMJ [53], and may contribute to the initiation of various phenomena, such as increased friction, adhesive forces, and shear stress [54, 55]. A compromised lubrication generates various levels of friction between the articular surfaces. The production of non-reducing disk phenomena and degenerative articular surface alterations that may also cause the onset of bone marrow edema and CE may be triggered by long-term mild friction [56–59].

The preventive use of MRI to identify subtypes of TMJ ID and OA may be suggested by this research [60, 61]. Early MRI diagnosis of disk displacement without reduction, bone marrow edema, and potentially associated erosive condylar processes can become an important factor in prevention and early treatment as it may prevent changes in dentofacial morphology or restricted mandibular development [41, 42]. In view of the fact that etiology, prognostic aspects, and treatment implications are the main aspects for the utility of diagnostic classifications [60], ongoing research is necessary to determine how well specific MRI findings of ID, OA and inflammatory TMJ alterations may demonstrate differences in pathogenesis, treatment, and prognosis.

Prognostic prediction models guide physicians upon therapeutic management and have become a standard to aid clinical decision-making. Demographics, clinical, and imaging characteristics, and specific test results are applied to derive these models, thereby estimating the probability of developing a particular outcome [62, 63]. In the development phase, multivariable regression techniques are used in model development [64]. In the validation phase, patient data not included in the development process are applied to test the developed model. With regard to the prognostic prediction model developed in the present study, it is therefore advised that external validation is performed in ongoing studies before considering to incorporate this model into clinical practice.

The present study may have failed to adequately assess the involved chronic TMJ pain patients. Chronic pain is considered a multidimensional emotional and sensory experience comprising affective, sensorial, and cognitive-evaluative aspects [65, 66]. The current study was limited by addressing only the sensory discriminative dimension of pain, i.e. further studies may have to apply multidimensional instruments to assess the chronic pain condition [6, 66]. Another limitation concerns the aspect that in studies assessing differences between study groups, the ideal control group should be the least symptomatic and the study sample should be the most diseased available. The TMJ arthralgia group may have contained too much "noise" by including TMJs sides with signs of associated palpation pain of the ipsilateral masticatory muscles, i.e., in some cases myalgia may have mimicked TMJ arthralgia [67]. Ongoing studies that involve a wellness "TMJ arthralgia" group characterized by the absence of associated signs of muscle palpation pain should be encouraged.

Observer bias from the radiologist may tend to have a significant impact on the assessment of MRI variables. Various factors such as quality of an image, specific criteria for interpretation, and training can affect observer performance. The current study used MRI images of high quality and applied well-defined criteria in the assessment of MRI variables, while intraobserver reliability was strong for the diagnostic criteria applied. However, study designs of medical imaging should routinely include more than one observer [68], i.e., consequently, overestimation of the significance of some MRI items to the described TMJ arthralgia groups may have occurred.

## Conclusion

The established prediction model using the risk factors of TMJ arthralgia may be useful for predicting the risk of CE. The data suggest MRI indicators as dominant factors in the definition of CE. Further research is needed to improve the model, and confirm the validity and reliability of the model.

## Data Availability

All data generated or analyzed during this study are not publicly available due to ethical and confidentiality reasons. The data will only be shared in aggregate form as presented in the figures and tables.

## Abbreviations

AUC: Area under the curve
CE: Condylar erosion
DDwoR/wLO: Disk displacement without reduction with limited opening
MRI: Magnetic resonance imaging
OA: Osteoarthritis
OR: Odds ratio
RDC/TMD: Research diagnostic criteria for temporomandibular disorders
TMJ: Temporomandibular joint
